# Antibacterial activity of antipsychotic agents, their association with lipid nanocapsules and its impact on the properties of the nanocarriers and on antibacterial activity

**DOI:** 10.1371/journal.pone.0189950

**Published:** 2018-01-03

**Authors:** Hassan Nehme, Patrick Saulnier, Alyaa A. Ramadan, Viviane Cassisa, Catherine Guillet, Matthieu Eveillard, Anita Umerska

**Affiliations:** 1 MINT, UNIV Angers, INSERM 1066, CNRS 6021, Université Bretagne Loire, Angers, France; 2 Department of Pharmaceutics, Faculty of Pharmacy, Alexandria University, Alexandria, Egypt; 3 Laboratoire de bactériologie, CHU Angers, France; 4 Service Commun de Cytometrie et d’analyse Nucleotidique (SCCAN), IFR 132, IBS–CHU, Angers, France; 5 Equipe ATIP AVENIR, CRCINA, Inserm, Université de Nantes, Université d'Angers, Angers, France; 6 Université de Lorraine, CITHEFOR, EA, Nancy, France; Universitatsklinikum Munster, GERMANY

## Abstract

Bacterial antibiotic resistance is an emerging public health problem worldwide; therefore, new therapeutic strategies are needed. Many studies have described antipsychotic compounds that present antibacterial activity. Hence, the aims of this study were to evaluate the *in vitro* antibacterial activity of antipsychotics belonging to different chemical families, to assess the influence of their association with lipid nanocapsules (LNCs) on their antimicrobial activity as well as drug release and to study the uptake of LNCs by bacterial cells. Antibacterial activity was evaluated against Gram-positive *Staphylococcus aureus* and Gram negative *Escherichia coli*, *Pseudomonas aeruginosa*, *Klebsiella pneumoniae* and *Acinetobacter baumannii* by minimum inhibitory concentration (MIC) assay, and the capability of killing tested microorganisms was evaluated by time kill assay. LNCs were prepared by phase inversion method, and the antipsychotic agents were incorporated using pre-loading and post-loading strategies. Only phenothiazines and thioxanthenes showed antibacterial activity, which was independent of antibiotic-resistance patterns. Loading the nanocarriers with the drugs affected the properties of the former, particularly their zeta potential. The release rate depended on the drug and its concentration—a maximum of released drug of less than 40% over 24 hours was observed for promazine. The influence of the drug associations on the antibacterial properties was concentration-dependent since, at low concentrations (high nanocarrier/drug ratio), the activity was lost, probably due to the high affinity of the drug to nanocarriers and slow release rate, whereas at higher concentrations, the activity was well maintained for the majority of the drugs. Chlorpromazine and thioridazine increased the uptake of the LNCs by bacteria compared with blank LNCs, even below the minimum inhibitory concentration.

## Introduction

Despite the abundance of antibiotics on the pharmaceutical market, their use has become increasingly limited due to the emergence of multidrug resistance among the microorganisms. The reason underlying this accelerated drug resistance has mostly been attributed to the abuse and misuse of antibiotics, bringing back the threat of bacterial infections many years after the first patients were cured [1]. Consequently, researchers have been focusing their work on finding new approaches to overcome this worldwide crisis. Some of them have devoted their efforts to the development of new semi-synthetic molecules to overcome specific bacterial resistance mechanisms by modulating the structures of existing antibiotics [2,3], while others have synthetized customized molecules for new bacterial targets [4]. Studies in this domain have brought to light that numerous compounds, belonging to different pharmacological families, bear significant antibacterial activity. Examples of such “non-antibiotics” include antihistamines, such as promethazine [5] and fluphenazine [6], antipsychotic compounds, such as promazine, chlorpromazine [7], thioridazine [8], trifluoperazine [9] and triflupromazine [10], anti-inflammatory agents, such as diclofenac [11], and even antihypertensive agents, such as methyl-DOPA [12] and propranolol [13]. To date, antipsychotic and antihistamine agents from the group of phenothiazines have been studied most extensively for their antimicrobial action both *in vitro* and *in vivo* [14]. Drug repurposing is now considered as an interesting approach to overcome the drawbacks of conventional antibiotics. Some of the key benefits of repositioning existing drugs to new indications include reduced time, cost and risk, as well as providing the means for safer and more effective treatments to be made available to patients. On the other hand, antipsychotic drugs are increasingly used in the population. By exerting antibacterial effects these drugs can cause selective pressure on bacteria and they could also promote antimicrobial resistance. It has been demonstrated that taking neuroleptic or antidepressant drugs was a risk factor for MRSA carriage [15].

Inadequate drug delivery to infected sites can lead to a low drug concentration at the target sites compared with that in the blood, which can be considered as a limitation of systemic drug delivery [16]. Moreover, sustained release of the drug might be desired to maintain high local concentrations for long periods of time without reaching systemic toxic concentrations [17]. Accordingly, nanomedicine could be considered as one of the most appropriate approaches for such cases since it allows for targeted delivery and prolonged release. Furthermore, in the case of lipophilic compounds, nanocarriers offer the possibility to obtain colloidal dispersions without using co-solvents. Another important advantage of these systems is that their solubilization capacity is retained on administration, in contrast to formulations based on conventional solubilization approaches [18,19]. In fact, using nanocarriers has already been adopted to enhance the activity of conventional antibiotics *in vitro* and *in vivo* [20].

Considering the high affinity of antipsychotic compounds to lipids [21], lipid-based nanocarriers offer an attractive delivery system for these drugs. Lipid nanocapsules (LNCs) are biomimetic nanocarriers used for the encapsulation of a broad variety of active ingredients. LNCs have numerous advantages such as good physical stability and manufacturability via a phase inversion temperature method, which is a low energy, organic solvent-free process [22,23]. LNCs contain an oily core composed of medium chain triglycerides and a surfactant shell made of a PEGylated surfactant and optionally a co-surfactant (usually lecithin). By varying the quantities of LNC ingredients (oil and surfactants) and by replacing or adding a co-surfactant and/or oil, the physicochemical and biological properties of the LNCs can be modified to obtain a delivery system optimized for a particular active ingredient and route of administration [24–28].

Based on these findings, this study was conducted to determine the *in vitro* antibacterial activity of 20 antipsychotic drugs belonging to different chemical classes against both reference and clinical strains of 5 bacterial species. Then, the six most active antipsychotics were associated with LNCs using different strategies, and their association efficiency and influence on the properties of the LNCs were examined and the *in vitro* drug release was studied. Another important purpose of this study was to evaluate the influence of association with the LNCs on the antibacterial activity of the drugs. Finally, the uptake of the LNCs by bacteria was assessed based on the fluorescence of DiO-labelled blank and drug-loaded LNCs.

## Materials and methods

### Materials

Antipsychotic compounds of different groups: an indol alkaloid reserpine, first generation typical antipsychotic drugs such as phenothiazines (chlorpromazine hydrochloride, fluphenazine dihydrochloride, perphenazine, prochlorperazine dimaleate salt, promazine hydrochloride, promethazine hydrochloride, thioridazine hydrochloride, trifluoperazine dihydrochloride, triflupromazine hydrochloride, and trimeprazine tartrate), thioxanthenes (chlorprothixene hydrochloride, cis-(Z)-flupenthixol dihydrochloride), butyrophenon (haloperidol), benzamide ((±)-sulpiride) and second generation atypical antipsychotic drugs (aripiprazole, clozapine, olanzapine, quetiapine hemifumarate salt, risperidone) were purchased from Sigma-Aldrich (France). Labrafac^®^ WL1349 (caprylic/capric acid triglycerides) was kindly provided by Gattefossé S.A. (France). Lipoid^®^ S75-3 (soybean lecithin) was kindly provided by Lipoïd Gmbh (Germany). Solutol^®^ HS15 (macrogol 15 hydroxystearate, polyoxyl 15 hydroxystearate; CAS number: 70142-34-6; molecular weight 963.24 g/mol; HLB 14–16) was kindly provided by BASF (Germany). Brain-heart infusion (BHI) broth was bought from bioMérieux (France). Plates containing Columbia agar supplemented with sheep blood were obtained from Oxoïd (France). DiO (DiOC_18_ (3), 3,3’-dioctadecyloxacarbocyanine perchlorate) was purchased from Thermo Fisher Scientific (France). All of the other solvents and chemicals were of analytical grade.

### Preparation of LNCs

Blank LNCs were produced at a concentration of 111mg/ml as described elsewhere [28]. The components of the LNCs: polyoxyl 15 hydroxystearate (846 mg), triglycerides (1028 mg), soybean lecithin (75 mg) and NaCl (90 mg) were weighted and mixed with 3 ml of water, heated to 90°C and cooled to 60°C. Three heating-cooling cycles were performed and during the last cooling cycle at the phase inversion temperature (80–83°C), the system was diluted with 12.5 ml of cold water.

To prepare chlorpromazine-loaded LNCs using a pre-loading strategy, the drug was weighed and mixed with other LNC ingredients before the addition of water and heating-cooling cycles.

To prepare drug-loaded LNCs using a post-loading strategy, the drugs were added to the blank LNC dispersions and incubated at 37°C for 1 h, which was adequate to attain dynamic equilibrium between the drugs associated with the LNCs and those in the surrounding medium.

To obtain DiO-labelled LNCs with a DiO concentration of 1 mg per gram of LNCs, 100 μl of DiO solution in acetone were added before the last cooling cycle.

### Characterization of LNCs

Dynamic light scattering (DLS) with 173° backscatter detection was used to determine the Z-average particle diameter and the polydispersity index (PDI) of the LNCs. Zeta potential was calculated after conversion of the electrophoretic mobility values, measured by laser Doppler velocimetry (LDV) adopting the Smoluchowski equation. Zetasizer nano series Nano-ZS fitted with a helium-neon laser performing at 633 nm (Malvern Instruments, UK) was used to obtain both the DLS and LDV measurements. The analysis was performed at an LNC concentration of 1.85 mg/ml obtained after 60-fold dilution of the initial LNCs dispersion with Milli-Q water. Each investigation was performed at 25°C in triplicate.

### Drug loading studies

Non-associated drugs were separated from the LNCs using a combined ultrafiltration-centrifugation technique involving Amicon^®^ Ultra-15 filters with a molecular weight cut-off of 10 kDa (Millipore, USA), as described elsewhere [27]. 2 ml of sample were placed in the sample reservoir (donor phase) of the centrifugal device and were centrifuged for 30 minutes at 4000 g. The solution in the filtrate vial (acceptor phase) was then weighed and analysed for drug content by HPLC, as described below. The volume of the sample remaining in the filter device (containing associated drug) was increased to 2 ml with deionized water. Next, the LNC dispersion from the sample reservoir was diluted at least 20-fold with methanol to rupture the particles and to extract the associated drug, which was quantified by HPLC.

The association efficiency (AE) and drug loading (DL) were determined using the following equations:

$$EE=\left[\frac{A-B}{A}\right]*100\%$$ (Eq 1)

where A is the total amount (mass) of the drug, and B is the mass of the non-associated drug; and

$$DL=\left[\frac{\left(A-B\right)}{C}\right]*100\%$$ (Eq 2)

where C is the total weight of all of the components of the nanoparticles (the associated drug and the mass of surfactants and oil used for the preparation of the LNCs).

### *In vitro* release studies

The *in vitro* release of the antipsychotic compounds was investigated using phosphate buffered solution (PBS) (137 mM NaCl, 2.7 mM KCl, 1.4 mM NaH_2_PO_4_, 1.3 mM Na_2_HPO_4_ adjusted to pH = 7.4 with NaOH solution) as a release medium. Aliquots of 200 μl of the sample were introduced into 15 ml polypropylene tubes containing 1.8 ml of PBS. The samples were then incubated at 37°C at 100 rpm in a reciprocal shaking water bath. At predetermined time intervals, samples were withdrawn and placed in the Amicon^®^ Ultra-15 centrifugal filters with a molecular weight cut-off of 10 kDa, and the released drug was separated by centrifugation for 10 minutes at 4000 g. After centrifugation, the solution from the filtrate vial (acceptor phase) was weighed, and the filtrate was assayed for drug content by HPLC. The LNC dispersion from the sample reservoir was standardized to 2 ml with PBS and was returned to a water bath to continue the release studies.

The release study data were fitted to a first-order equation:

$$W=W_{\infty }(1-e^{-kt})$$ (Eq 3)

where W is the amount of drug released at time t (based on cumulative release), W_∞_ is the amount of drug released at infinity, and k is the release rate constant [29].

### Quantification of antipsychotic agents by HPLC

The drug contents were analyzed using an HPLC system previously described [25]. Briefly, standard solutions of the drugs (0.1–1000 μg/ml) were prepared in deionized water, and 20 μl of the standard or sample were injected into the SymmetryShield ^TM^ RP_18_ 5 μm 4.6x250 mm column (Waters, USA). A flow rate of 1.0 ml/min was employed using mobile phase A composed of 0.1% trifluoroacetic acid (TFA) in water, and mobile phase B, composed of 0.1% TFA in acetonitrile. A linear gradient was run: 20% B for 25 min, 45% B for 0.1 minute, 100% B for 4.9 minutes, 20% B for 0.1 minute, and 20% B for 14.9 minutes. The drug peaks had retention times of approximately 27.7 minutes for chlorpromazine, 28.4 minutes for chlorprothixene, 23.6 minutes for promazine, 31.0 minutes for thioridazine, 23.7 minutes for trifluoperazine and 30.1 minutes for trifluperazine. UV detection was performed at 200 nm. Empower®3 software was used for data collection and integration.

The method showed good linearity for all of the tested molecules (R^2^≥0.999). The quantification limits were 1 μg/ml for chlorpromazine, chloprothixene, promazine and triflupromazine and 2 μg/ml for thioridazine and trifluoperazine. More than 85% of drug was recovered each time.

### Bacterial strains

Antibacterial activity was tested against five reference strains: *Staphylococcus aureus* (ATCC 25923), *Pseudomonas aeruginosa* (ATCC 27853), *Escherichia coli* (ATCC25922), *Acinetobacter baumannii* AYE (ATCC BAA-1710) and *Klebsiella pneumoniae* (DSM 16609); and five clinical isolates: methicillin-resistant *Staphylococcus aureus* (MRSA) (0702E0196), *Pseudomonas aeruginosa* (0704C0134), extended-spectrum beta-lactamase (ESBL) *Escherichia coli* (9007550201), *Acinetobacter baumannii* RCH and extended-spectrum beta-lactamase (ESBL) *Klebsiella pneumoniae* (16510661801). The clinical isolates were acquired from the University Hospital of Angers (France). Before starting the experiments, the bacteria were grown overnight on Columbia agar supplemented with sheep blood at 37°C.

### Determination of minimum inhibitory concentration (MIC)

A broth microdilution method was used to determine the MIC. To obtain the desired concentration range, serial two-fold dilutions of the samples in BHI were prepared. The microorganism suspension in 0.85% NaCl with optical density equal to that of the 0.5 McFarland standard was diluted 10 -fold with BHI medium. 50 μl of bacterial suspension in BHI broth were then added individually into each well of a sterile 96-well plate that already contained 50 μl of the tested sample or control. The positive control wells included only BHI and the bacterial suspension, whereas the negative control wells included exclusively BHI and the tested sample. The plates were finally incubated at 37°C for 24 hours. MIC tests were performed in triplicates on separate days. The MIC was defined as the lowest drug concentration that completely inhibited the visible bacterial growth. MIC values were considered different if they varied by more than one dilution.

### Time-kill assay

The microorganism suspension in 0.85% NaCl with optical density equal to that of the 0.6 McFarland standard was diluted 10 -fold with BHI medium. The samples were diluted to a volume of 1.98 ml with BHI broth and 20 μl of bacterial suspension were then added to each polypropylene tube. Bacterial suspension in BHI broth without tested formulation/compound was considered a control. The final suspensions were incubated at 37°C. Serial 100-fold dilutions were prepared in distilled water with 100 μl of suspension withdrawn from each tube after 0, 3, 6 and 24 hours. An overall amount of 100 μl of diluted and/or undiluted sample was transferred onto the agar surface and was spread gently to be well absorbed into the agar. The agar plates were incubated overnight at 37°C, and the colonies were eventually counted. Time-kill experiments were performed in triplicates on separate days.

### Flow cytometry

The bacterial suspension in 0.85% NaCl with optical density equal to that of the 1.0 McFarland standard was diluted 10 -fold with BHI medium. The samples were diluted to a volume of 9.9 ml with BHI broth, and 100 μl of bacterial suspension were then added to each polypropylene tube. The microorganisms were incubated for 10 minutes at 37°C with DiO-labelled blank, thioridazine- and chlorpromazine-loaded LNCs at concentrations corresponding to 2 and 0.5 MICs. The bacteria were collected by centrifugation (3000 g, 10 minutes) and suspended in PBS. Two washing cycles were performed. All of the analyses were performed using a MACSquant^®^ flow cytometer (Miltenyi Biotec, France). The instrument was set up to measure the size (forward scatter), granularity (side scatter) and bacterial cell fluorescence. DiO uptake was measured by analysing individual cells for fluorescence. The mean fluorescence intensity was determined after correction for cell auto-fluorescence, and fluorescence histograms were obtained for 5000 individual events. The data were analysed using the MACSQuantify™ software and are expressed as percentages of control fluorescence in arbitrary units. All of the experiments were conducted at least 3 times.

### Statistical analysis

The statistical significance of the differences between samples was determined using one-way analysis of variance (ANOVA). Differences were considered significant at p < 0.05.

## Results and discussion

### *In vitro* antibacterial properties of antipsychotic drugs

To date, research has focused on the antibacterial activity of phenothiazine derivatives. However, there are many antipsychotic drugs on the market characterized by different structures and mechanisms of action. Therefore, the first aim of this paper was to examine whether they exhibit antibacterial effects. For that reason, a wide range of antipsychotic drugs, including typical and atypical antipsychotic agents, reserpine and some phenothiazine derivatives used mainly as antihistamines, was selected. The MIC values of the tested compounds that displayed antibacterial activity are shown in Table 1.

**Table 1:** Minimum inhibitory concentrations (MICs) of antipsychotic agents against different bacteria. MICs are expressed in μg/ml.

	SA ATCC	MRSA	PA ATCC	PA clinical	E Coli ATCC	ESBL E coli	AB ATCC	AB RCH	KP DSM	ESBL KP
Chlorpromazine	64	64	256	1024	64	64	64	64	64–128	64–128
Chlorprothixene	64	64	1024	≥1024	64	64	64	64	128–256	512–1024
Flupenthixol	32	32	>1024	>1024	512	1024	64	64–128	1024	≥1024
Fluphenazine	64	64	>1024	>1024	128	256	128	128	512	512
Perphenazine	512	512	>1024	>1024	1024	>1024	1024	1024	>1024	>1024
Prochlorperazine	128–256	128–256	>1024	>1024	512	512	512	512	1024	1024
Promazine	128	128	512	512	128	128	128	128	128	128
Promethazine	128	128	512–1024	1024	128	128	128	128	128–256	128–256
Thioridazine	32	32	≥1024	>1024	128	128	128	128	256	256
Trifluoperazine	32	32	≥1024	≥1024	64	128	64	64	256–512	256
Triflupromazine	64	32–64	1024	1024	64	64	64	64	128	128–256
Trimeprazine	128	128	≥1024	≥1024	128	128–256	128–256	256	256	256

SA: *Staphylococcus aureus*, MRSA: methicillin-resistant *Staphylococcus aureus*, PA: *Pseudomonas aeruginosa*, E. coli: *Escherichia coli*, ESBL: extended-spectrum beta-lactamase, AB: *Acinetobacter baumannii*, KP: *Klebsiella pneumoniae*

None of the investigated atypical antipsychotic compounds (aripiprazole, clozapine, olanzapine, quetiapine, risperidone), reserpine, haloperidol (butyrophenon) and sulpiride (benzamide) showed antibacterial against tested bacterial strains (MIC>1024 μg/ml; not shown). Phenothiazines and thioxanthenes showed activity with variable MIC results, depending on the bacterial strains. There were similarities in the antibacterial activity between the groups since their MIC values were not different by more than one serial dilution, which was observed by comparing chlorpromazine with chlorprothixene and fluphenazine with flupentixol. Those compounds had MIC values of 32–64 μg/ml against Gram-positive *Staphylococcus aureus* and 64–128 μg/ml against Gram-negative bacteria, such as *Escherichia coli*, *Acinetobacter baumannii* and *Klebsiella pneumoniae*. This resemblance could be explained by, in both chemical classes, the compared drugs having the same molecular structure with the only difference of a double bond to the side chain that replaces the nitrogen atom at position 10 of the b ring facing the sulphur atom at position 5 of the phenothiazine ring in the case of the thioxanthene group. In the phenothiazines group, perphenazine was the least active in all cases with an MIC ≥ 1024 μg/ml against Gram-negative strains and 512 μg/ml against Gram-positive *Staphylococcus aureus*. Prochlorperazine also showed considerably less activity than other phenothiazines. Flupenthixol displayed strong activity against *Staphylococcus aureus* and *Acinetobacter baumannii*, but *Pseudomonas aeruginosa*, *Klebsiella pneumoniae* or *Escherichia coli* showed little or no sensitivity to this compound. Other phenothiazines showed similar activity with MIC values between 32 and 128 μg/ml against *Staphylococcus aureus* and 64–256 μg/ml against *Escherichia coli* and *Acinetobacter baumannii*. Promazine, the most hydrophilic compound, exhibited broad spectrum antibacterial activity with the smallest variations in MIC values between all of the tested strains (128–512 μg/ml). Antibacterial activity is not correlated with antipsychotic activity. For instance, the equivalent doses of oral antipsychotics were 100 mg for chlorpromazine and 5 mg for trifluoperazine (50 -fold difference), whereas the variation in MIC of these compounds was much smaller (0–4 -fold depending on the strain). Interestingly, the antibacterial effects of phenothiazines and thioxanthens were independent of the antibiotic resistance. Gram-positive *Staphylococcus aureus* strains were the most sensitive to the tested drugs since their MIC was 32–64 μg/ml, except for prochlorperazine, trimeprazine, promazine and promethazine, which were slightly less active, with an MIC of 128–256 μg/ml. Gram-negative strains were generally more resistant to antipsychotic drugs than *Staphylococcus aureus*. The target for antipsychotic drugs is probably the cytoplasmic membrane, which is present in both, Gram-negative and Gram-positive bacteria [14]. To reach this target, the molecules have to penetrate the bacterial envelope, which has different structure in Gram-negative and Gram-positive bacteria. Although the peptidoglycan layer in Gram-positive bacteria is thicker than in Gram-negative bacteria, the former do not contain the outer membrane. The difference in sensitivity to antipsychotic agents is due to the barrier properties of the outer membrane of Gram-negative bacteria, which is an asymmetric phospholipid bilayer into which specific uptake channels and unspecific porins are embedded. Thus, the penetration of hydrophilic drugs is size -limited by narrow pores [30,31]. In the outer leaflet, lipopolysaccharides (LPS) are anchored by their lipid A fractions, while their polar polysaccharide moieties are projected to the outside of the surface [32]. The inner core of the LPS forms a pseudo-static gel-like structure, caused by the hydrocarbons chains, which slows the penetration of hydrophobic molecules [33]. This finding explains the higher MIC values in the case of Gram-negative strains since all of the tested phenothiazine and thioxanthenes derivatives are hydrophobic. The moderate influx of both hydrophilic and hydrophobic compounds across the outer membrane is additionally antagonized by active efflux transporters dispersed within the inner membrane and interacting with other components of the periplasmic space and the outer membrane to form trans-envelope complexes [34]. Among all of the investigated bacterial strains, both clinical and reference strains of *Pseudomonas aeruginosa* were the most resistant to antipsychotics, with chlorpromazine, promethazine and promazine being the most active, having an MIC ranging from 256 μg/ml to 1024 μg/ml. This finding could be explained by the synergistic action of the very low permeability of this bacterium’s outer membrane, acquired by the use of slow porins instead of the classical trimeric porins, and by the multidrug efflux systems that are characterized by their broad substrate specificity and their constitutive expression [35].

The MICs of all tested antipsychotic agents were considerably lower than those of conventional antibiotics (usually below 1–2 μg/ml). Additionally, the MICs of antipsychotics are higher than physiological concentrations of these drugs. High MIC values limit their potential use as antibacterial agents on their own. Nonetheless it is important to examine different aspects of interactions of antipsychotic agents with bacteria, because they can be harmful to patients by increasing the selection pressure in the microorganisms. When the microorganisms are exposed to sublethal concentrations of antipsychotic agents the resistance to these compounds can potentially develop. However, both these drawbacks (i.e. high MIC values and possibility to develop resistance) can be overcome by using antipsychotic drugs in combination with other antimicrobial agents. Indeed, many phenothiazines have shown synergistic interactions with several antibiotics thereby lowering the doses administered to patients [14].

**Fig 1:**
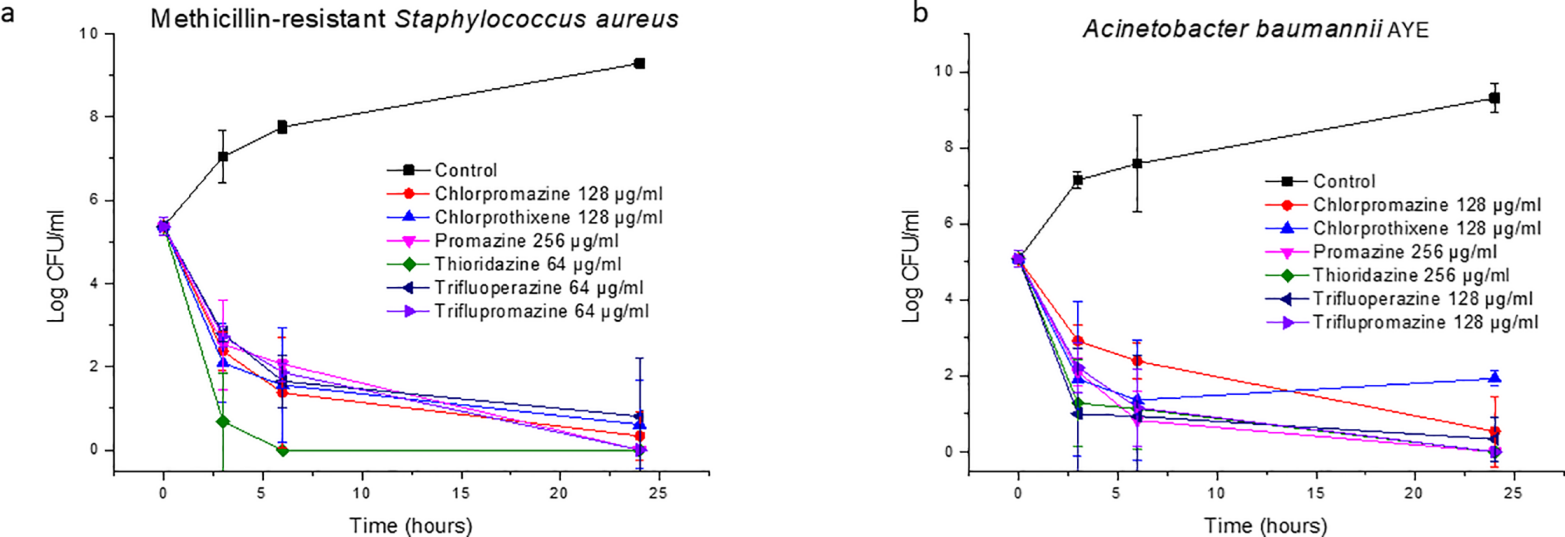
**Time-kill curves of antipsychotic drugs against (a) methicillin-resistant *Staphylococcus aureus* and (b) *Acinetobacter baumannii* AYE (mean ± SD, n = 3)**.

The time-kill curves of antipsychotic compounds are shown in Fig 1. The concentrations used for this assay were equal to 2 MIC of each corresponding drug. Generally, all of the tested drugs had a bactericidal activity against *Staphylococcus aureus* and *Acinetobacter baumannii* since the number of colonies was reduced by more than 3 Log in both cases. A sharp decrease in CFU number was observed within three hours of incubation, in good agreement with previously reported data [36]. Phenothiazines affect the components of plasma membrane, such as efflux pumps, energy sources and energy providing enzymes (e.g., ATPase). They also have an influence of genes that code for the permeability aspect of a bacterium [14]. Rapid bactericidal action of antipsychotic drugs could be the result of their effects on the plasma membrane.

### Properties of drug-loaded LNCs

The characteristics of the lipid nanocapsules, prepared using pre-loading and post-loading strategy, are shown in Tables 2 and 3, respectively. LNC formulations containing chlorpromazine at 0.5 and 2 mg/ml were successfully obtained using the pre-loading strategy. Nevertheless, after an increase in the chlorpromazine concentration to 8 mg/ml, the LNCs could not be obtained because the phase inversion did not occur, and aggregation and phase separation were observed. Higher concentrations, such as 33 mg/ml, were easily achieved by using the post-loading strategy. The possibility of achieving high drug concentrations is therefore an important advantage of the post-loading strategy. Moreover, post-loading could also be the preferred option for drugs sensitive to high temperatures because, in this approach, the drug does not undergo heating-cooling cycles. Generally, most of the antipsychotic compounds were efficiently associated with the LNCs since they presented an association efficiency greater than 90% at all of the tested concentrations. The association was slightly affected by the drug’s concentration in the cases of chlorpromazine, chlorprothixene, thioridazine and triflupromazine since it remained greater than 90% at 33 mg/ml of drug, although a significant reduction in association efficiency was observed. However, the association efficiency was significantly decreased from 99% and 95% to 68.9% and 49.3% when the drug’s concentration increased from 0.5 mg/ml to 33 mg/ml in the cases of trifluoperazine and promazine, respectively. Interestingly, there was no difference in the association efficiency of chlorpromazine between the pre- and post-loading strategies since they both presented similar association efficiency values of 96% and 93% at concentrations of 0.5 mg/ml and 2 mg/ml of chlorpromazine, respectively. In addition, the drug loading of almost all of the tested formulations increased proportionally to the increasing drug’s concentration and reached approximately 21% at 33 mg/ml, apart from trifluoperazine and promazine, which reached, at the same concentration, drug loading of only 15.7% and 11.2%, respectively due to their lower association efficiency. High drug loading is desirable since it enables achieving therapeutic concentrations and reduces the concentration of the nanocarrier, thereby minimizing possible toxic effects [37]. Indeed, the drug concentrations that could be achieved in LNC formulations, e.g., for chlorpromazine, were higher than in the manufactured products, indicating that this level of association is satisfactory.

**Table 2:** Properties of blank and drug-loaded lipid nanocapsules prepared using a pre-loading strategy.

Chlorpromazine concentration (mg/ml)	AE (%)	DL (%)	PS (nm)	PDI	ZP (mV)
0 (blank LNCs)	N/A	N/A	52.5±1.8	0.038±0.008	-5.81±0.99
0.512	96.8±0.28	0.443±0.001	53.5±1.8	0.042±0.018	+2.41±1.28***
2.048	93.4±0.64	1.687±0.012	75.2±4.1***	0.133±0.011***	+11.5±0.79***

AE: association efficiency, DL: drug loading, PS: particle size, PDI: polydispersity index, ZP: zeta potential.

*p<0.05

**p<0.01

***p<0.001

**Table 3:** Properties of drug-loaded lipid nanocapsules prepared using a post-loading strategy.

Drug concentration (mg/ml)	AE (%)	DL (%)	PS (nm)	PDI	ZP (mV)
Chlorpromazine					
0.512	96.1±1.63	0.440±0.007	53.7±1.1	0.045±0.016	-0.03±0.70**
2.048	93.0±1.77	1.679±0.032	77.2±5.5**	0.123±0.014***	+11.77±1.85***
8.192	90.1±2.76	6.173±0.189	55.2±1.3	0.075±0.005**	+22.53±3.15***
32.768	89.4±2.19	20.324±0.498	58.7±2.6*	0.062±0.023	+34.7±3.42***
Trifluoperazine					
0.512	99.0±0.10	0.453±0.001	54.4±1.8	0.045±0.013	+4.22±2.51**
2.048	90.5±1.06	1.634±0.019	55.7±3.1	0.059±0.025	+17.80±2.82***
8.192	74.8±2.33	5.125±0.160	53.2±1.0	0.043±0.005	+29.67±3.75***
32.768	68.9±2.69	15.663±0.612	54.2±0.2	0.051±0.023	+33.50±3.87***
Thioridazine					
0.512	99.5±0.10	0.455±0.001	54.5±0.3	0.046±0.001	+2.43±2.10**
2.048	98.7±0.35	1.782±0.006	75.3±2.0***	0.120±0.004***	+18.00±1.13***
8.192	98.0±0.21	6.715±0.014	54.9±2.2	0.043±0.003	+30.67±5.40***
32.768	94.1±3.04	21.392±0.691	55.7±1.9	0.046±0.001	+30.60±4.20***
Chlorprothixene					
0.512	98.7±0.57	0.452±0.003	53.4±0.3	0.054±0.018	+0.19±2.23*
2.048	95.4±0.42	1.723±0.008	74.0±1.9***	0.124±0.006***	+13.85±3.89**
8.192	91.6±0.21	6.276±0.014	56.7±2.7	0.062±0.016	+28.4±7.14**
32.768	91.3±0.92	20.756±0.209	57.9±2.0*	0.084±0.008*	+34.45±3.67***
Promazine					
0.512	95.0±1.48	0.435±0.007	53.2±1.1	0.032±0.008	-3.01±0.71*
2.048	87.9±0.99	1.587±0.018	55.6±0.2*	0.085±0.032	+5.40±1.31***
8.192	73.4±2.83	5.029±0.194	71.0±2.4***	0.113±0.008***	+14.30±0.40***
32.768	49.3±7.21	11.208±1.639	61.5±2.8**	0.067±0.014*	+25.00±2.10***
Triflupromazine					
0.512	98.2±0.10	0.449±0.001	54.4±1.9	0.062±0.028	+1.98±5.60
2.048	97.5±0.20	1.761±0.004	74.5±2.3***	0.111±0.007***	+15.20±1.27***
8.192	95.1±0.35	6.516±0.024	54.1±1.9	0.057±0.013	+25.37±2.15***
32.768	94.3±0.67	21.438±0.152	59.5±2.2*	0.067±0.016*	+29.00±5.09***

AE: association efficiency, DL: drug loading, PS: particle size, PDI: polydispersity index, ZP: zeta potential.

*p<0.05

**p<0.01

***p<0.001

Generally, apart from promazine, there was no significant difference in particle size between blank and drug-loaded LNCs at drug concentrations of 0.5, 8 and 33 mg/ml. Interestingly, at a drug concentration of 2 mg/ml, the particle size significantly increased from approximately 50 nm to 70 nm. In the case of promazine, the particle size increased from 53 nm to 71 nm when the drug concentration increased from 0.5 to 8 mg/ml and then decreased to 62 nm at a drug concentration of 33 mg/ml. There was no difference in the particle size between the two loading strategies since the particle size increased from 53 to 75–77 nm when the drug’s concentration increased from 0.5 to 2 mg/ml in the case of chlorpromazine-loaded LNCs for both the pre- and post-loading strategies. There was no important difference in the polydispersity index of the LNCs, reflecting the homogeneous size distribution of the nanocapsules.

In all cases, the LNCs became positively charged after being loaded with the antipsychotic compounds. Their zeta potential increased proportionally to the increase in the drug concentration from a negative value of –5.81 mV for blank LNCs to positive values that reached a maximum of +34.77 mV in the case of chlorpromazine-loaded LNCs. Similar behaviour (i.e., an increase in zeta potential corresponding to increasing concentrations of associated molecules) was observed for peptide-loaded LNCs [27]. These changes in zeta potential could be explained by, the antipsychotic drugs, which are positively charged due to the amino groups in their structure and, consequently, high pKa, being located on the surface of the lipid nanocapsules, thereby changing the surface charge from negative to positive. Theoretically, the adsorption of antipsychotic drugs on LNCs could be driven by electrostatic interactions between amino groups of drugs and phosphate groups of lecithin, dipole-ion interactions between a negative dipole on PEG molecules and a positive charge on drug molecules and hydrophobic interactions, similar to previously described amphiphilic peptides [27]. We performed additional experiments with adsorption on LNCs composed solely of macrogol 15 hydroxystearate and triglycerides, without lecithin, and the association efficiency values were similar to those observed for the LNCs containing lecithin (not shown). Therefore, it could be concluded that the interactions between lecithin molecules and antipsychotic drugs are not important for drug adsorption and that the adsorption is driven by dipole-ion interactions and hydrophobic interactions between the drugs and components of the LNCs. Interestingly, the adsorption of antipsychotic drugs was much higher than that observed for peptides on lecithin -LNCs under similar conditions (17–33%) [27], perhaps because of the peptide-resistant character of PEG moieties caused by steric stabilization effects. The molecules of antipsychotic drugs are much smaller than those of peptides; therefore, the steric stabilization effects are less important.

### *In vitro* drug release

**Fig 2:**
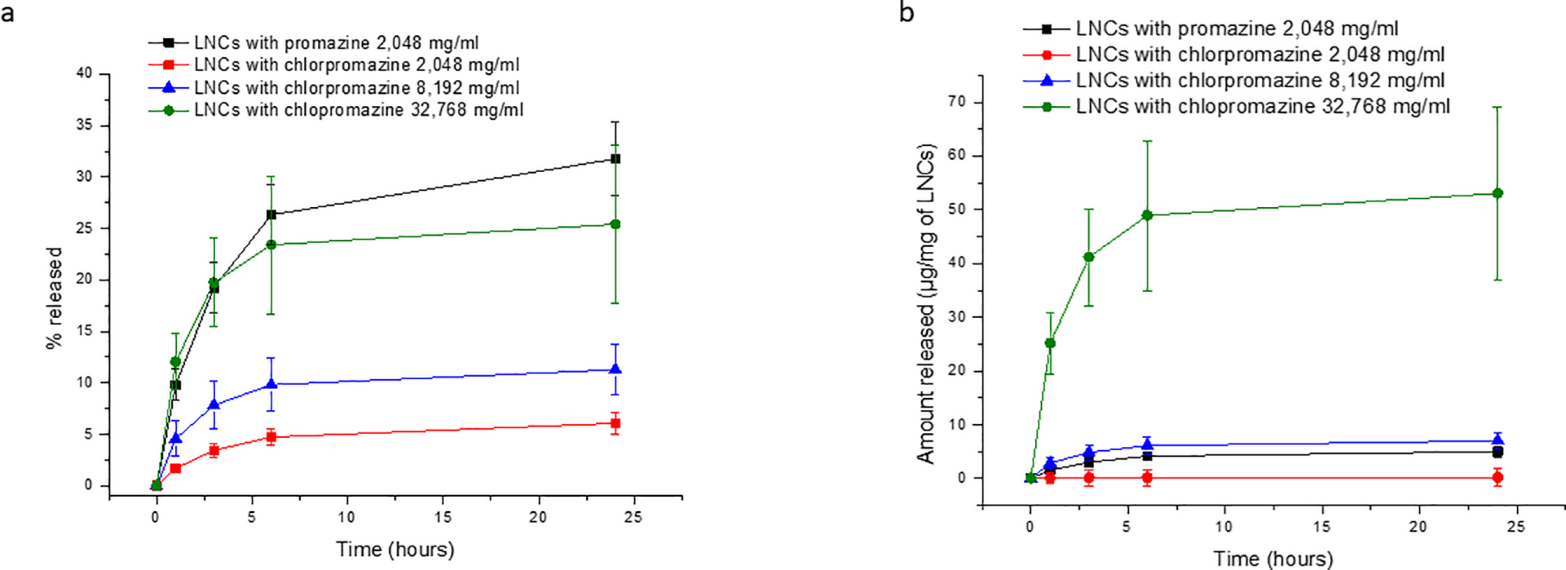
**Cumulative release profiles of chlorpromazine and promazine from drug-loaded LNCs, expressed as a percentage of drug released (A) and amount of drug released per mg of LNCs (B)**.

This *in vitro* release study of antipsychotic compounds from the LNCs was performed to: (i) confirm the success of the drugs’ associations; (ii) understand the release kinetics and mechanisms; and (iii) determine the effect of the drugs’ concentration on the release profile. Among the six encapsulated antipsychotic compounds, only promazine and chlorpromazine were released from the LNCs (Fig 2), whereas no drug was discharged in the cases of thioridazine, trifluoperazine, triflupromazine and chlorprothixene (not shown). The retention of the antipsychotic drugs in the LNCs could be explained by the high affinity of these hydrophobic compounds to the LNCs, possibly due to surface activity of the drugs and their interactions with the components of the core and shell of the LNCs, which oppose drug diffusion outside the LNCs. The release of chlorpromazine and promazine from the LNCs followed first order kinetics and the obtained parameter estimates and related statistics are shown in Table 4.

**Table 4:** Model parameter estimates for chlorpromazine and promazine release fitted to the first-order model (Eq 3).

Drug	Drug concentration	k (h^-1^)	W_∞_ (μg/mg of LNCs)	Goodness of fit (R^2^)
Promazine	2.048	0.333±0.010	4.9±0.53	0.9964
Chlorpromazine	2.048	0.281±0.007	1.0±0.17	0.9953
Chlorpromazine	8.192	0.450±0.107	6.8±1.42	0.9901
Chlorpromazine	32.768	0.627±0.120	51.5±15.49	0.9930

W_∞_: the amount of drug released at infinity;k: the release rate constant

Generally, all of the formulations showed a sustained release profile that reached a maximum amount of released drug less than 40% after 24 hours. Interestingly, the drug release rate of chlorpromazine increased proportionally with the increase in the drug’s concentration in the LNCs, as illustrated by good linear correlation (R^2^ = 0.901) between the drug concentration and release rate constant. Indeed, the increased drug density at the LNC surface, due to the increase in the drug concentration in the formulation, led eventually to the rapid release of the fraction that presents the weakest interactions with the LNC components. Furthermore, the drug release rate of promazine at a concentration of 2 mg/ml was significantly higher (p = 0.0018) than that of chlorpromazine at the same concentration. This finding could be explained by promazine being less hydrophobic than any of the tested antipsychotic compounds and thus being readily released from the lipid nanocapsules. An important advantage is that no burst effect was observed. Therefore, LNCs could be a promising delivery system for antipsychotic drugs because they combine the advantages of prolonged release and the small size of the nanocarriers, which could be easily administered via different routes, such as intravenously.

### Antibacterial properties of drug-loaded LNCs

The MIC values of the antipsychotic drugs loaded in the LNCs are shown in Table 5. None of the preparations with drug concentrations of 0.5 mg /ml and 2 mg/ml showed activity against any of the tested bacterial strains (not shown).

**Table 5:** Minimum inhibitory concentrations (MICs) of antipsychotic agents loaded in the LNCs against different bacteria. MICs are expressed in μg/ml.

Drug concentration (mg/ml)	SA ATCC	MRSA	PA ATCC	PA clinical	E Coli ATCC	ESBL E coli	AB ATCC	AB RCH	KP DSM	ESBL KP
Chlorpromazine										
8.192	≥512	512	>512	>512	256–512	512	256–512	256	>512	>512
32.768	128	64	>512	>512	128	128	64–128	64–128	128–256	256
Chlorprothixene										
8.192	>512	>512	>512	>512	>512	>512	>512	>512	>512	>512
32.768	128	64	>512	>512	512	128	256	256	>512	>512
Promazine										
8.192	256	128–256	>512	>512	128	128	256	128	256	256
32.768	256	128	512	>512	128	128	128	128	128	256
Thioridazine										
8.192	64	32	>512	>512	>512	>512	>512	>512	>512	>512
32.768	32	16	>512	>512	512	512	128–256	256	>512	>512
Trifluoperazine										
8.192	>512	>512	>512	>512	>512	>512	>512	>512	>512	>512
32.768	64	32	>512	>512	512	>512	>512	>512	>512	>512
8.192	>512	>512	>512	>512	>512	>512	>512	>512	>512	>512
32.768	128	64	>512	>512	256	512	256–512	256	>512	>512

SA: *Staphylococcus aureus*, MRSA: methicillin-resistant *Staphylococcus aureus*, PA: *Pseudomonas aeruginosa*, E coli: *Escherichia coli*, ESBL: extended-spectrum beta-lactamase, AB: *Acinetobacter baumannii*, KP: *Klebsiella pneumoniae*

The preparations with drug concentrations of 8 mg/ml showed mostly reduced or no antibacterial activity, apart from thioridazine and promazine, which had the same antibacterial activity against *Staphylococcus aureus* as the free drugs since their MIC values were 32–64 and 128–256 μg/ml, respectively. The preparations that were inactive against *Staphylococcus aureus* at concentrations of 8 mg/ml, such as trifluoperazine, chlorpromazine, chlorprothixene and triflupromazine, restored their antibacterial activity against these bacteria at concentrations of 33 mg/ml. This activity was identical to that of free drugs since their MIC values were identical or not different by more than one consecutive dilution. Apart from trifluoperazine and triflupromazine the activity of which was reduced, all of the other antipsychotics showed the same antibacterial activity against *Acinetobacter baumannii* and *Escherichia coli* as that of free drugs. Chlorprothixene showed reduced activity only against *Escherichia coli ATCC* since its MIC was increased from 64 μg/ml to 512 μg/ml after association.

*Pseudomonas aeruginosa* and *Klebsiella pneumoniae* were resistant to all of the preparations at all of the tested concentrations, apart from chlorpromazine, which had moderate activity against *Klebsiella pneumoniae* with an MIC value of 128–256 μg/ml, which was less than the non-associated drug, having an MIC of 64–128 μg/ml, and promazine since it had an MIC of 512 μg/ml against *Pseudomonas aeruginosa*.

Interestingly, promazine maintained its broad spectrum even after loading into lipid nanocapsules, and it showed the same activity against all of the tested strains at both concentrations (8 mg/ml and 33 mg/ml), with an MIC of 128–256 μg/ml, similar to that of the non-associated form, with the exception of *Pseudomonas aeruginosa*.

The decreased activity of drugs associated with the LNCs, observed particularly at lower concentrations, could be attributed to their high affinity to the nanocarriers. This finding was further confirmed by the *in vitro* release data. The release was correlated well with the antibacterial activity of the drugs. Promazine, which exhibited the fastest release rate and the largest quantity of drug released, showed the best preservation of antibacterial activity after associating with the LNCs. Chlorpromazine, which was also released more rapidly than 4 other drugs, showed relatively good preservation of its antibacterial activity. The increase in antibacterial activity, corresponding to the increased concentration of associated drug (decreased LNC/drug ratio), was also correlated well with the fastest release of larger quantities of drug observed at higher concentrations. Indeed, when the drug was presented to bacteria in small quantities for a prolonged time period, it could be more effectively neutralized by the microorganisms than with the same quantity delivered at once. In conclusion, drug release is an important parameter affecting the antibacterial activity of antipsychotic agents associated with LNCs.

Similar to our findings, loading into nanocarriers has been shown to affect the antibacterial activity of loaded compounds, such as antimicrobial peptides [38,39]. Interestingly, the employment of nanocarriers with antibacterial properties, such as monolaurin -LNCs, which showed potent activity against *Staphylococcus aureus*, resulted in synergistic interactions with peptides derived from plectasin [25], which is a promising strategy for other compounds such as antipsychotic drugs.

**Fig 3:**
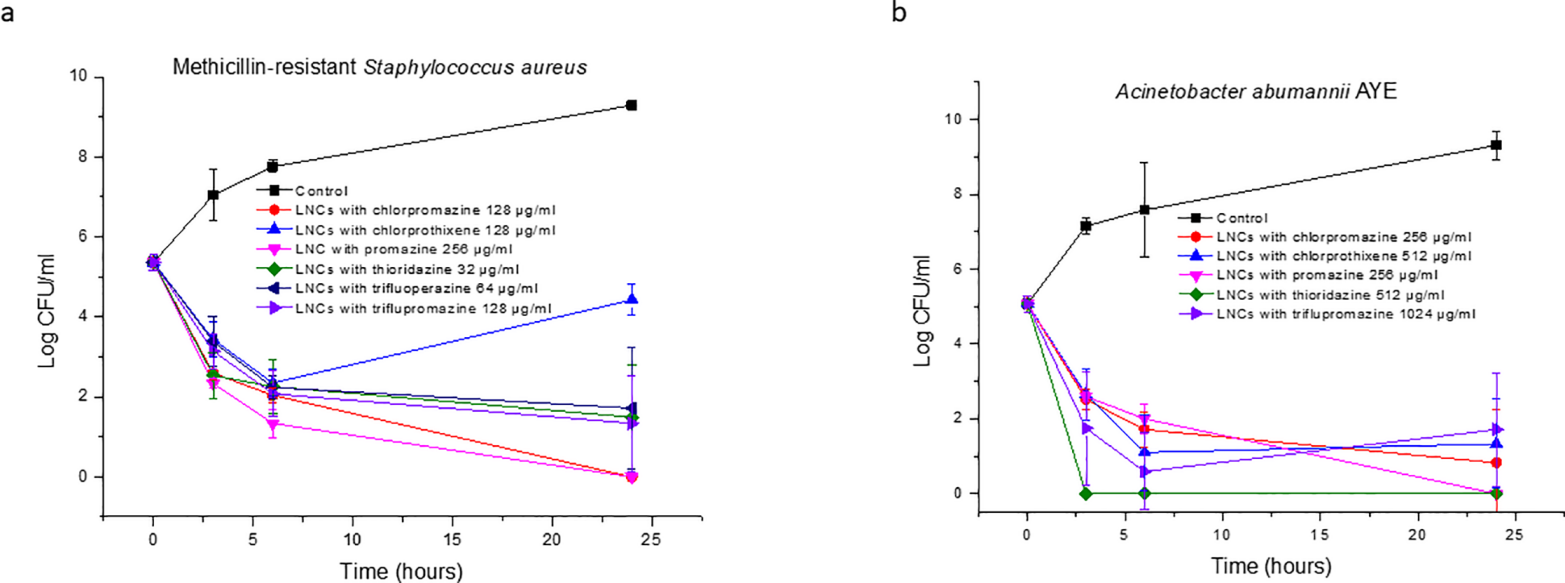
**Time-kill curves of antipsychotic drugs associated with the LNCs against (a) methicillin-resistant *Staphylococcus aureus* and (b) *Acinetobacter baumannii* AYE (mean ± SD, n = 3)**.

The time-kill curves of associated antipsychotic compounds are shown in Fig 3. The concentrations used in this assay were equal to 2 MIC of the drug associated with the LNCs. Similar to non-associated compounds, all of the drug-loaded preparations exerted bactericidal activity against both *Staphylococcus aureus* and *Acinetobacter baumannii*, with the number of colonies reduced by more than 3 Log.

### Influence of antipsychotic drugs on LNC uptake by *Staphylococcus aureus*

**Fig 4:**
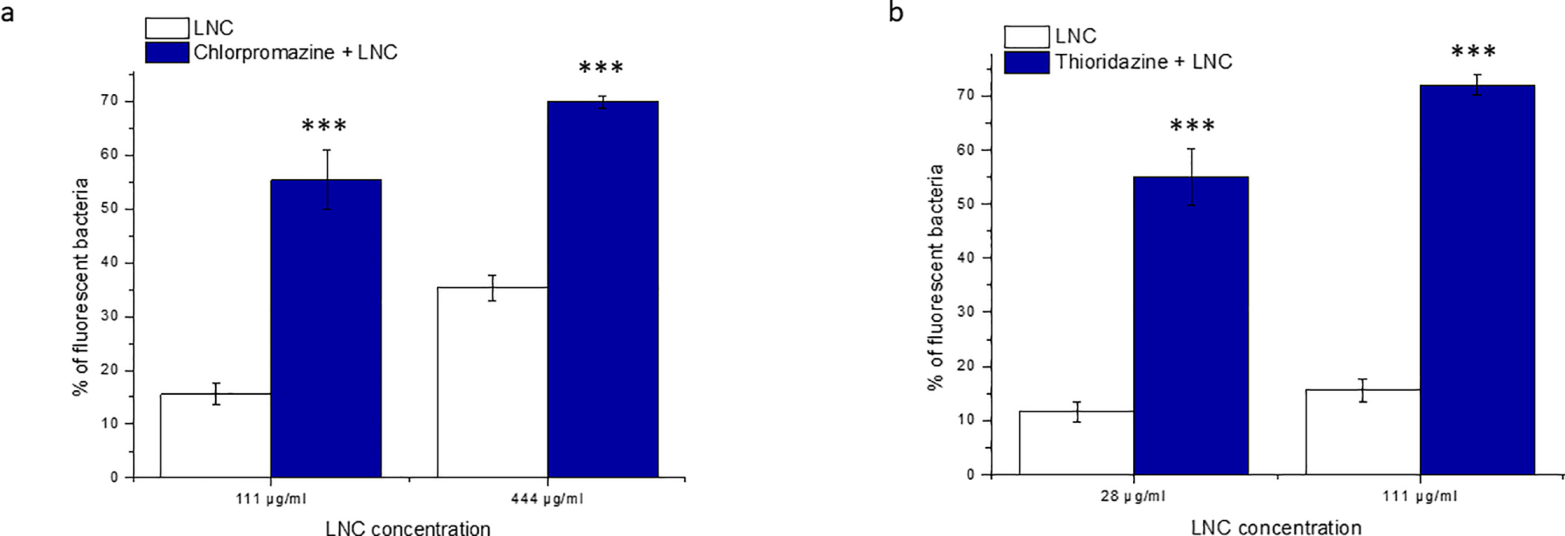
**Influence of the antipsychotic drugs (a) chlorpromazine and (b) thioridazine on LNC uptake by *Staphylococcus aureus*. The LNC/drug ratio was 3.4.** ***p < 0.001 versus blank LNCs.

Because antipsychotic drug-loaded LNCs showed potent activity against *Staphylococcus aureus*, the ability of blank, thioridazine- and chlorpromazine-loaded LNCs to be taken up by these bacteria was quantified by flow cytometry. The uptake of LNCs labelled with DiO was evaluated for blank and drug-loaded LNCs at concentrations corresponding to 0.5 and 2 MIC of drugs after 10 minutes of incubation with methicillin-resistant *Staphylococcus aureus*. Such a short incubation time was selected because of a rapid increase in bacterial number, which could affect the results in the cases of samples that did not inhibit bacterial growth. The percentage of fluorescent bacteria, reflecting the number of internalized LNCs, increased significantly from 11.6% to 35.3% when the LNC concentration changed from 28 μg/ml to 444 μg/ml (Fig 4). Similarly, the penetration of LNCs into the bacterial cells was increased after incorporation of drugs into the nanocarriers, compared with the blank LNCs at similar nanocarrier concentrations. At 0.5 MIC, the percentage of fluorescent bacteria reached 55% and 55.4% after incorporation of 8 μg/ml and 32 μg/ml of thioridazine and chlorpromazine, respectively, which was significantly higher than that of the blank LNCs at the same concentrations. Indeed, the association of the hydrophobic and positively charged antipsychotic compounds with the LNCs resulted in positively charged nanocarriers with increased affinity for the hydrophobic and negatively charged bacterial cell wall due to the enhanced electrostatic and van der Waals interactions between them. Moreover, the LNC uptake that was increased by antipsychotic drugs can be additional proof the stability of drug/LNC association. Interestingly, thioridazine improved the uptake of the LNCs further than did chlorpromazine since the percentage of fluorescent bacteria reached 72% when 32 μg/ml (2 MIC) of thioridazine was incorporated into 111 μg/ml of LNC, whereas it reached only 55.4% in the case of chlorpromazine at the same concentrations (0.5 MIC) and 69.9% at concentrations of 444 μg/ml and 128 μg/ml of LNCs and chlorpromazine (2 MIC), respectively. This difference could be explained by the higher hydrophobicity of thioridazine, thus its greater affinity to the bacterial cell. In summary, chlorpromazine and thioridazine increased the LNC uptake by *Staphylococcus aureus* even at concentrations less than the MIC. Moreover, the uptake was augmented by an increased drug concentration. Because LNCs can be used as carriers for antibiotics, the addition of antipsychotic drugs could facilitate the penetration of LNCs loaded with antibacterial molecules into bacterial cells, where they could exert their activity. Moreover, further studies of the uptake of antipsychotic drug-loaded LNCs by eukaryotic cells, such as macrophages, are needed because this delivery system could have potential applications in the treatment of intracellular infections.

## Summary and conclusions

Several interesting findings emerged from this study. Among the tested antipsychotic agents from different chemical groups, only phenothiazines and thioxanthenes showed antibacterial activity. They proved to be bactericidal and exerted their activity independently of antibiotic-resistance.

To obtain LNCs associated with antipsychotic drugs, the post-loading strategy proved to be better than pre-loading because the former enabled the obtaining of higher drug concentration and notably high drug loading. The properties of the LNCs, particularly the zeta potential, were affected by associated drug- an inversion from a negative to positive charge was observed with the zeta potential values, dependent on the amount of the associated drug, suggesting adsorption of the drug in the surface layer of the LNCs. LNCs constitute a promising delivery system for the antipsychotic drugs because they are capable of providing prolonged release of those compounds with a release rate that depended on the drug and its concentration. However, the high affinity for the nanocarriers and the prolonged release resulted in a loss of antibacterial activity at lower drug concentrations. To obtain drug-loaded LNCs with potent antibacterial activity, it is necessary to use high drug concentrations (low LNC/drug ratio). An increase in LNC uptake by bacterial cells after association with antipsychotic drugs might provide opportunities for synergistic interactions between these drugs and the antibacterial agents that could be potentially encapsulated in the LNCs.
